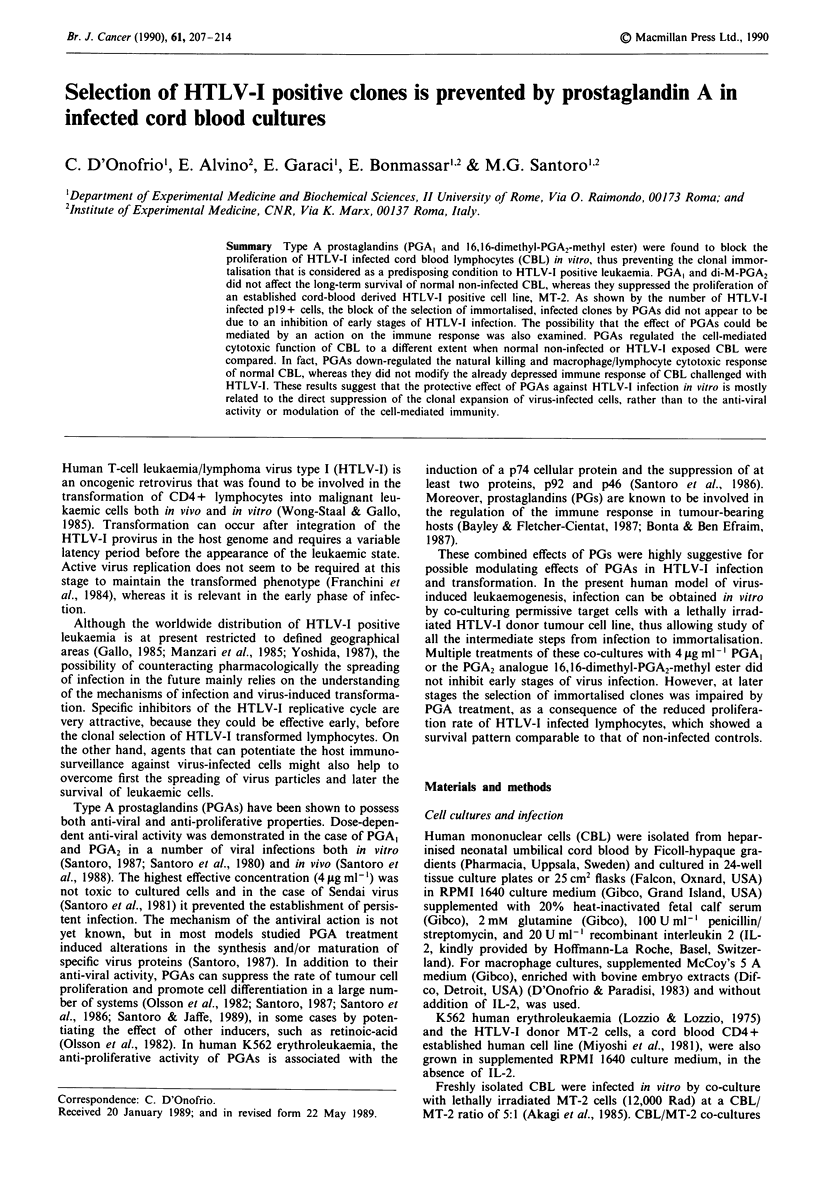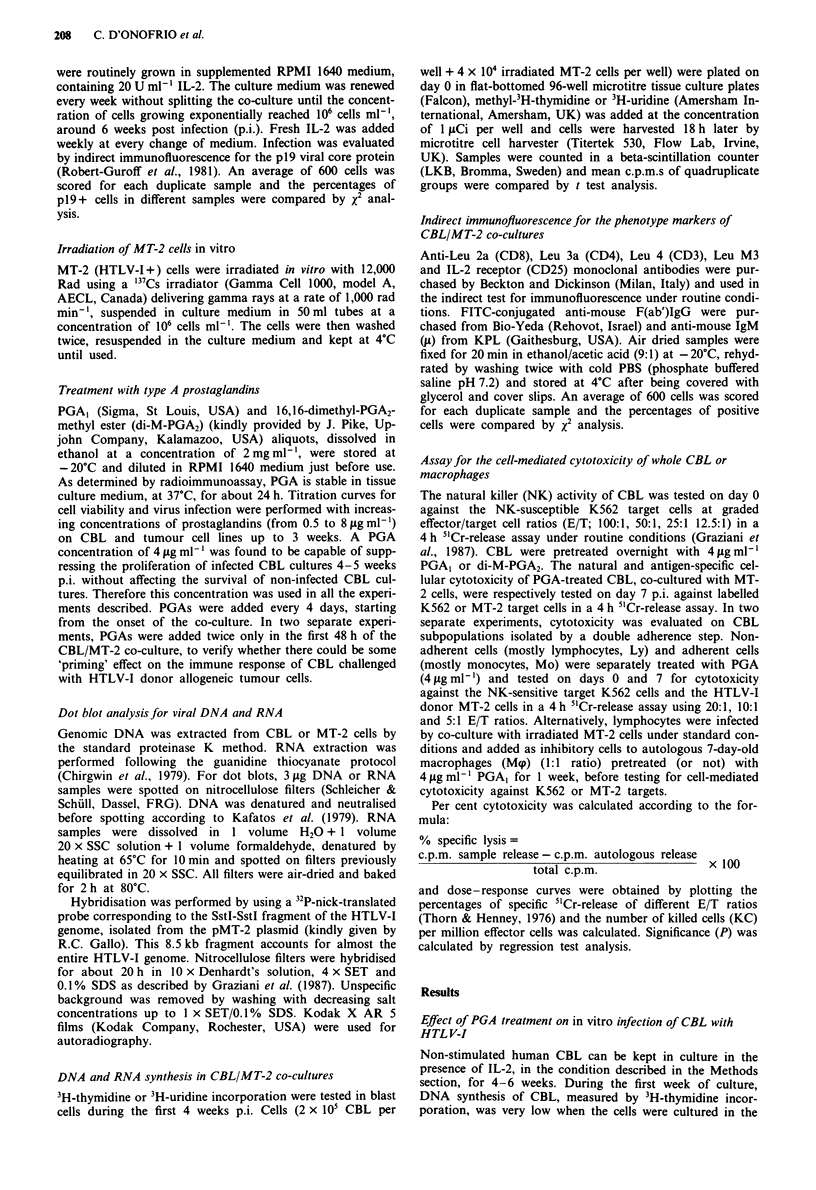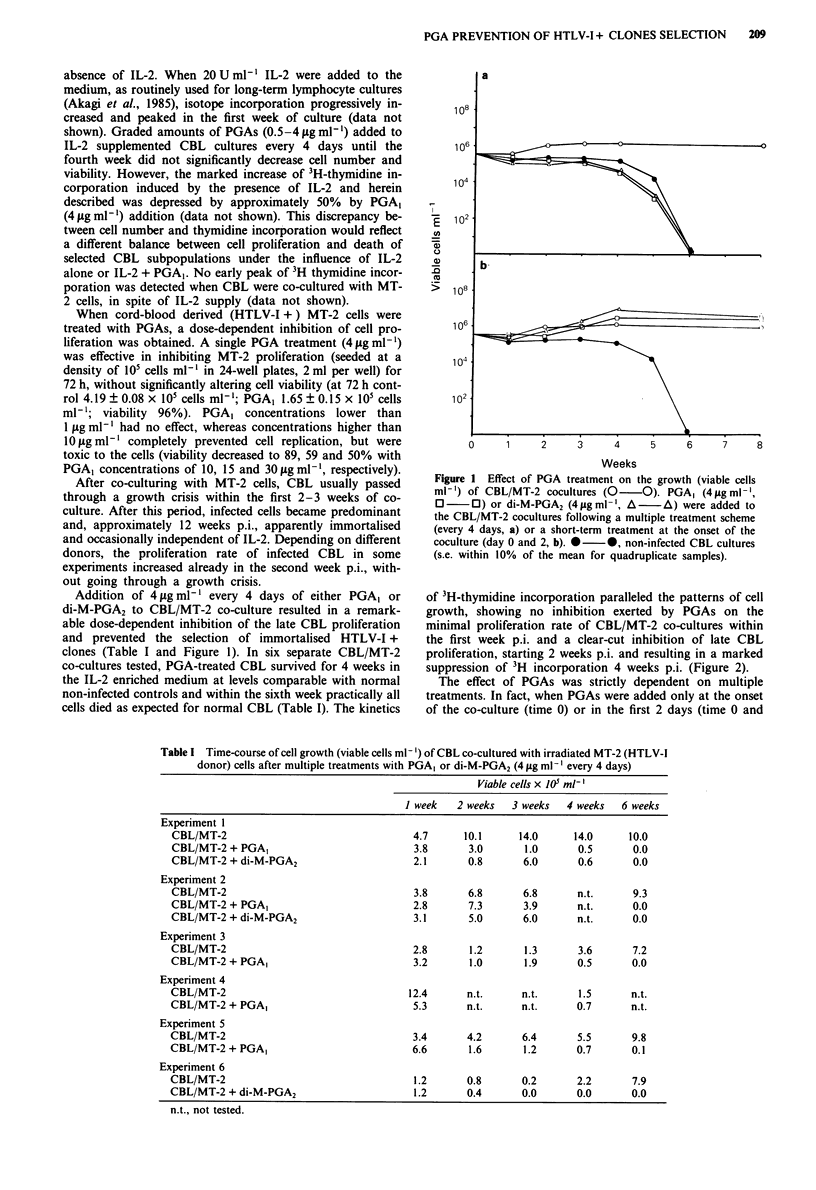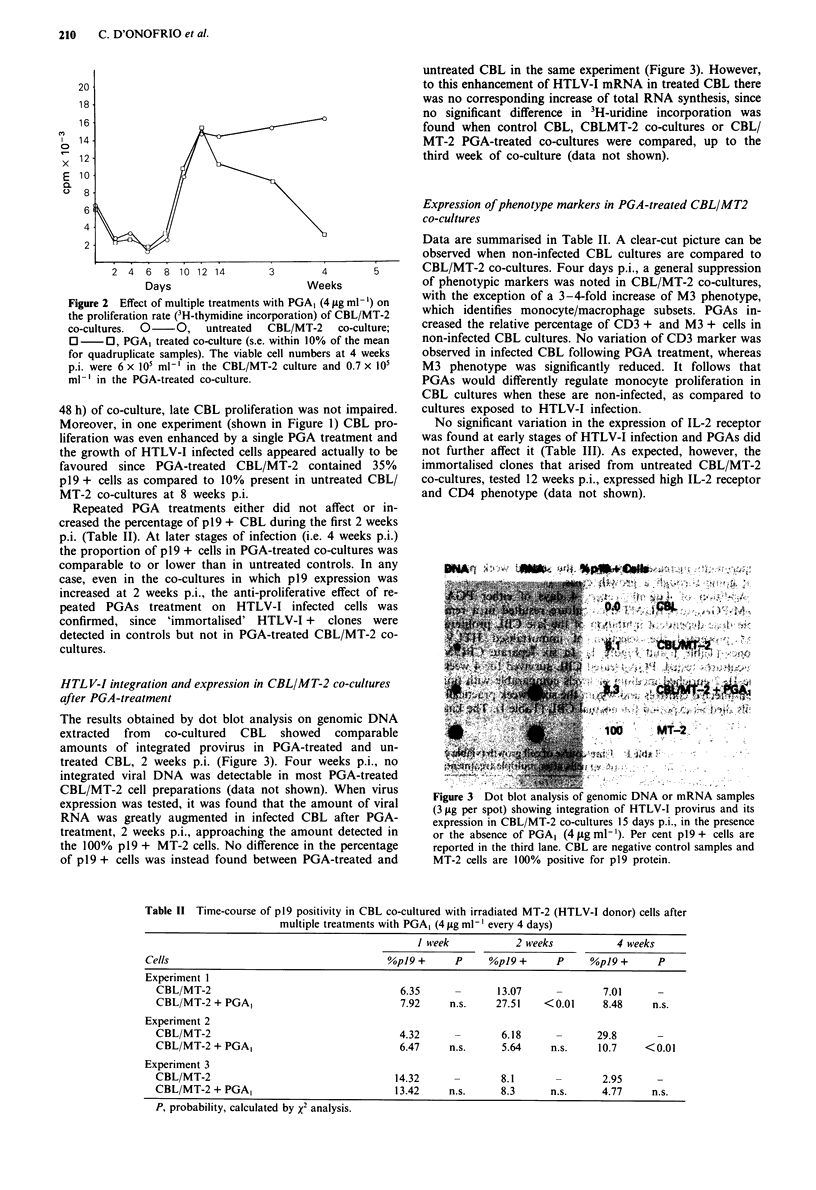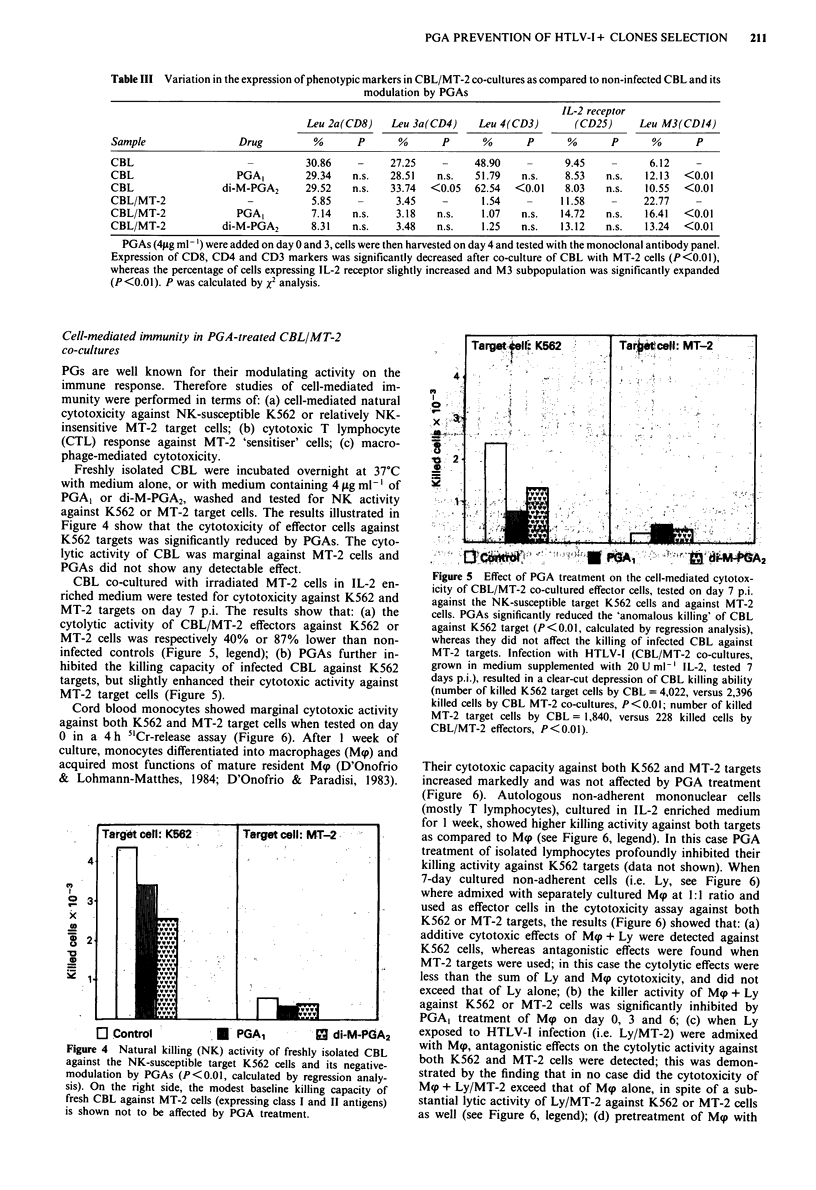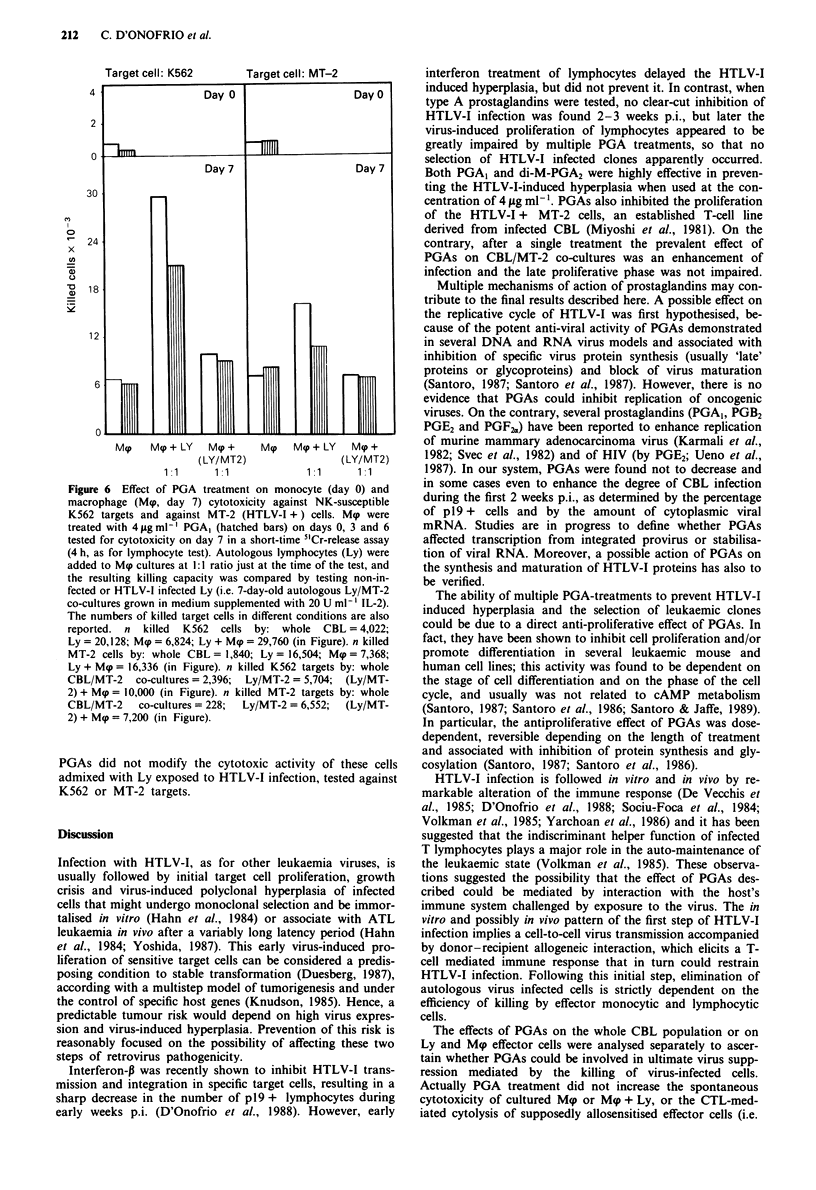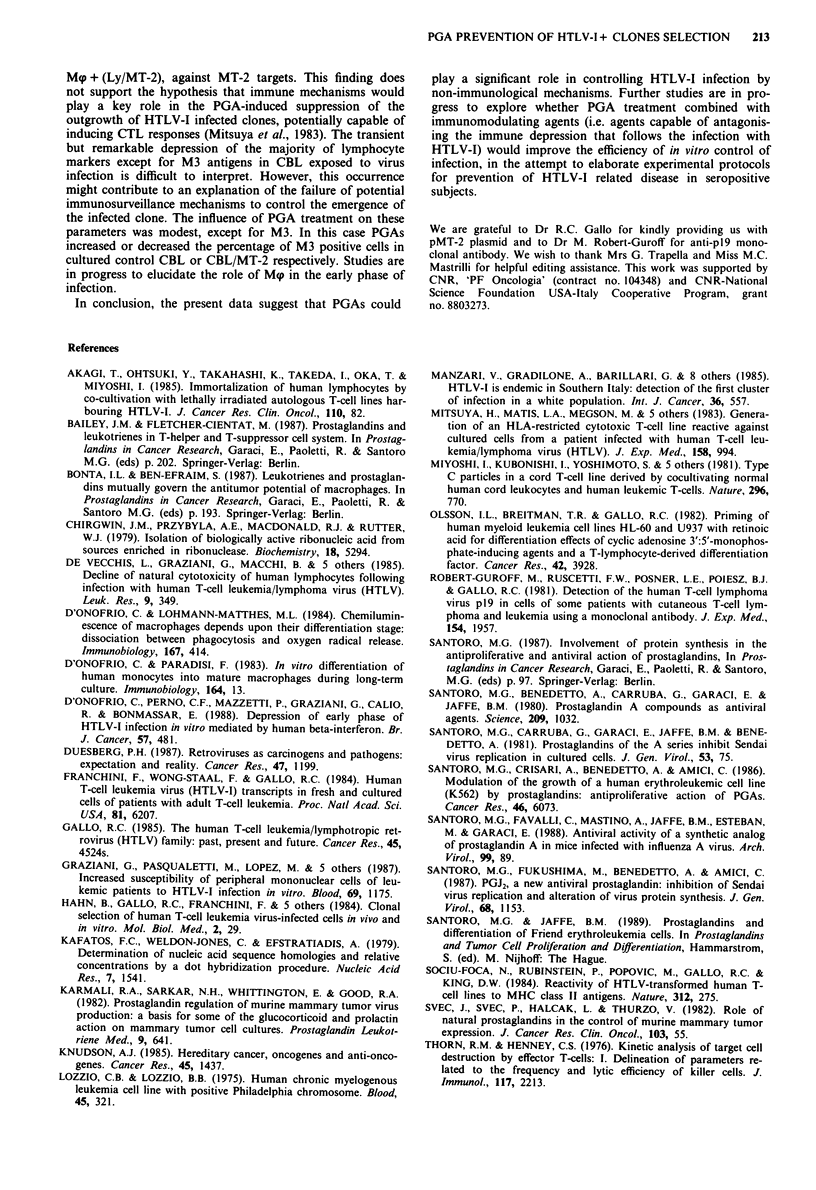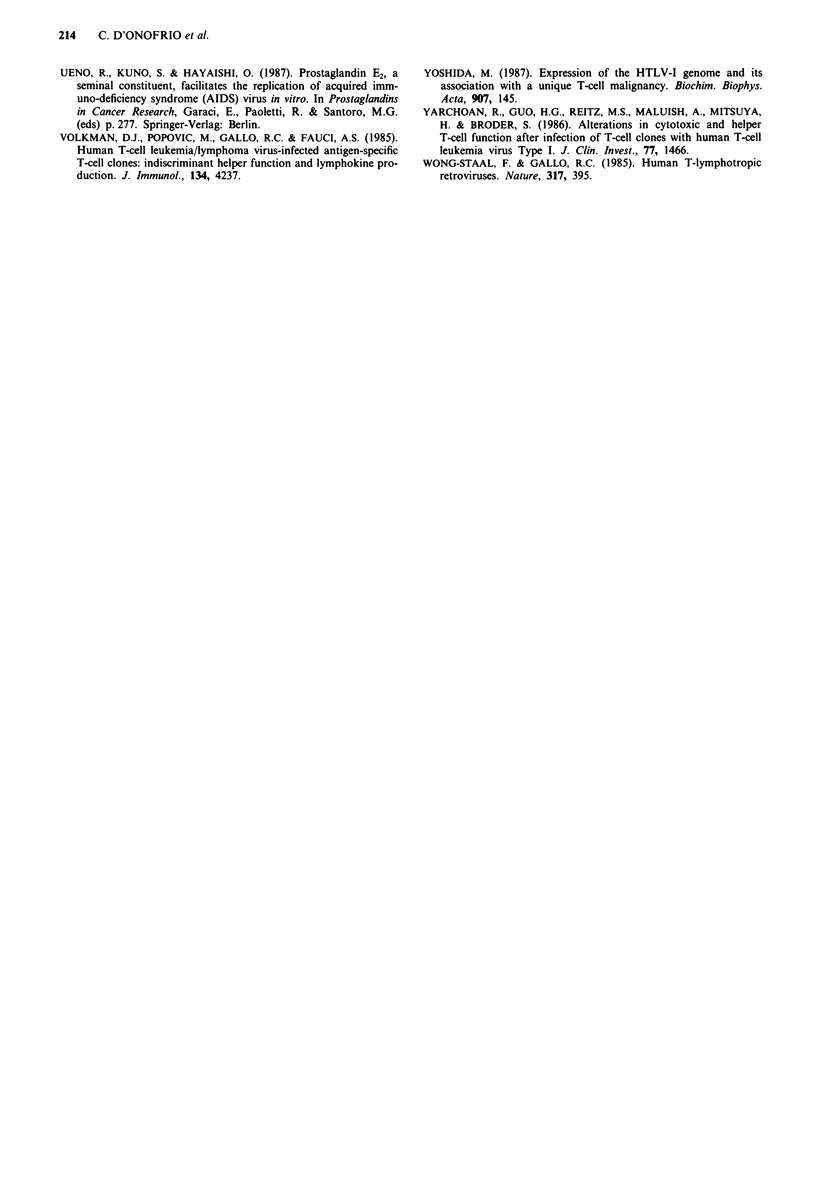# Selection of HTLV-I positive clones is prevented by prostaglandin A in infected cord blood cultures.

**DOI:** 10.1038/bjc.1990.38

**Published:** 1990-02

**Authors:** C. D'Onofrio, E. Alvino, E. Garaci, E. Bonmassar, M. G. Santoro

**Affiliations:** Department of Experimental Medicine and Biochemical Sciences, II University of Rome, Italy.

## Abstract

**Images:**


*Br. J. Cancer* (1990), **61**, 207–214 © Macmillan Press Ltd., 1990

### Selection of HTLV-I positive clones is prevented by prostaglandin A in infected cord blood cultures

C. D'Onofrio[1], E. Alvino[2], E. Garaci[1], E. Bonmassar[1,2] & M.G. Santoro[1,2]

[1]*Department of Experimental Medicine and Biochemical Sciences, II University of Rome, Via O. Raimondo, 00173 Roma; and* [2]*Institute of Experimental Medicine, CNR, Via K. Marx, 00137 Roma, Italy.*

**Summary** Type A prostaglandins ($PGA_1$ and 16,16-dimethyl-$PGA_2$-methyl ester) were found to block the proliferation of HTLV-I infected cord blood lymphocytes (CBL) *in vitro*, thus preventing the clonal immortalisation that is considered as a predisposing condition to HTLV-I positive leukaemia. $PGA_1$ and di-M-$PGA_2$ did not affect the long-term survival of normal non-infected CBL, whereas they suppressed the proliferation of an established cord-blood derived HTLV-I positive cell line, MT-2. As shown by the number of HTLV-I infected p19+ cells, the block of the selection of immortalised, infected clones by PGAs did not appear to be due to an inhibition of early stages of HTLV-I infection. The possibility that the effect of PGAs could be mediated by an action on the immune response was also examined. PGAs regulated the cell-mediated cytotoxic function of CBL to a different extent when normal non-infected or HTLV-I exposed CBL were compared. In fact, PGAs down-regulated the natural killing and macrophage/lymphocyte cytotoxic response of normal CBL, whereas they did not modify the already depressed immune response of CBL challenged with HTLV-I. These results suggest that the protective effect of PGAs against HTLV-I infection *in vitro* is mostly related to the direct suppression of the clonal expansion of virus-infected cells, rather than to the anti-viral activity or modulation of the cell-mediated immunity.

Human T-cell leukaemia/lymphoma virus type I (HTLV-I) is an oncogenic retrovirus that was found to be involved in the transformation of CD4+ lymphocytes into malignant leukaemic cells both *in vivo* and *in vitro* (Wong-Staal & Gallo, 1985). Transformation can occur after integration of the HTLV-I provirus in the host genome and requires a variable latency period before the appearance of the leukaemic state. Active virus replication does not seem to be required at this stage to maintain the transformed phenotype (Franchini *et al.*, 1984), whereas it is relevant in the early phase of infection.

Although the worldwide distribution of HTLV-I positive leukaemia is at present restricted to defined geographical areas (Gallo, 1985; Manzari *et al.*, 1985; Yoshida, 1987), the possibility of counteracting pharmacologically the spreading of infection in the future mainly relies on the understanding of the mechanisms of infection and virus-induced transformation. Specific inhibitors of the HTLV-I replicative cycle are very attractive, because they could be effective early, before the clonal selection of HTLV-I transformed lymphocytes. On the other hand, agents that can potentiate the host immunosurveillance against virus-infected cells might also help to overcome first the spreading of virus particles and later the survival of leukaemic cells.

Type A prostaglandins (PGAs) have been shown to possess both anti-viral and anti-proliferative properties. Dose-dependent anti-viral activity was demonstrated in the case of $PGA_1$ and $PGA_2$ in a number of viral infections both *in vitro* (Santoro, 1987; Santoro *et al.*, 1980) and *in vivo* (Santoro *et al.*, 1988). The highest effective concentration (4 $\mu g\ ml^{-1}$) was not toxic to cultured cells and in the case of Sendai virus (Santoro *et al.*, 1981) it prevented the establishment of persistent infection. The mechanism of the antiviral action is not yet known, but in most models studied PGA treatment induced alterations in the synthesis and/or maturation of specific virus proteins (Santoro, 1987). In addition to their anti-viral activity, PGAs can suppress the rate of tumour cell proliferation and promote cell differentiation in a large number of systems (Olsson *et al.*, 1982; Santoro, 1987; Santoro *et al.*, 1986; Santoro & Jaffe, 1989), in some cases by potentiating the effect of other inducers, such as retinoic-acid (Olsson *et al.*, 1982). In human K562 erythroleukaemia, the anti-proliferative activity of PGAs is associated with the induction of a p74 cellular protein and the suppression of at least two proteins, p92 and p46 (Santoro *et al.*, 1986). Moreover, prostaglandins (PGs) are known to be involved in the regulation of the immune response in tumour-bearing hosts (Bayley & Fletcher-Cientat, 1987; Bonta & Ben Efraim, 1987).

These combined effects of PGs were highly suggestive for possible modulating effects of PGAs in HTLV-I infection and transformation. In the present human model of virus-induced leukaemogenesis, infection can be obtained *in vitro* by co-culturing permissive target cells with a lethally irradiated HTLV-I donor tumour cell line, thus allowing study of all the intermediate steps from infection to immortalisation. Multiple treatments of these co-cultures with 4 $\mu g\ ml^{-1}$ $PGA_1$ or the $PGA_2$ analogue 16,16-dimethyl-$PGA_2$-methyl ester did not inhibit early stages of virus infection. However, at later stages the selection of immortalised clones was impaired by PGA treatment, as a consequence of the reduced proliferation rate of HTLV-I infected lymphocytes, which showed a survival pattern comparable to that of non-infected controls.

### Materials and methods

### *Cell cultures and infection*

Human mononuclear cells (CBL) were isolated from heparinised neonatal umbilical cord blood by Ficoll-hypaque gradients (Pharmacia, Uppsala, Sweden) and cultured in 24-well tissue culture plates or 25 $cm^2$ flasks (Falcon, Oxnard, USA) in RPMI 1640 culture medium (Gibco, Grand Island, USA) supplemented with 20% heat-inactivated fetal calf serum (Gibco), 2 mM glutamine (Gibco), 100 U $ml^{-1}$ penicillin/streptomycin, and 20 U $ml^{-1}$ recombinant interleukin 2 (IL-2, kindly provided by Hoffmann-La Roche, Basel, Switzerland). For macrophage cultures, supplemented McCoy's 5 A medium (Gibco), enriched with bovine embryo extracts (Difco, Detroit, USA) (D'Onofrio & Paradisi, 1983) and without addition of IL-2, was used.

K562 human erythroleukaemia (Lozzio & Lozzio, 1975) and the HTLV-I donor MT-2 cells, a cord blood CD4+ established human cell line (Miyoshi *et al.*, 1981), were also grown in supplemented RPMI 1640 culture medium, in the absence of IL-2.

Freshly isolated CBL were infected *in vitro* by co-culture with lethally irradiated MT-2 cells (12,000 Rad) at a CBL/MT-2 ratio of 5:1 (Akagi *et al.*, 1985). CBL/MT-2 co-cultures

Correspondence: C. D'Onofrio.
Received 20 January 1989; and in revised form 22 May 1989.

were routinely grown in supplemented RPMI 1640 medium, containing 20 U $ml^{-1}$ IL-2. The culture medium was renewed every week without splitting the co-culture until the concentration of cells growing exponentially reached $10^6$ cells $ml^{-1}$, around 6 weeks post infection (p.i.). Fresh IL-2 was added weekly at every change of medium. Infection was evaluated by indirect immunofluorescence for the p19 viral core protein (Robert-Guroff et al., 1981). An average of 600 cells was scored for each duplicate sample and the percentages of p19+ cells in different samples were compared by $\chi^2$ analysis.

*Irradiation of MT-2 cells* in vitro

MT-2 (HTLV-I+) cells were irradiated *in vitro* with 12,000 Rad using a $^{137}Cs$ irradiator (Gamma Cell 1000, model A, AECL, Canada) delivering gamma rays at a rate of 1,000 rad $min^{-1}$, suspended in culture medium in 50 ml tubes at a concentration of $10^6$ cells $ml^{-1}$. The cells were then washed twice, resuspended in the culture medium and kept at 4°C until used.

*Treatment with type A prostaglandins*

$PGA_1$ (Sigma, St Louis, USA) and 16,16-dimethyl-$PGA_2$-methyl ester (di-M-$PGA_2$) (kindly provided by J. Pike, Upjohn Company, Kalamazoo, USA) aliquots, dissolved in ethanol at a concentration of 2 mg $ml^{-1}$, were stored at −20°C and diluted in RPMI 1640 medium just before use. As determined by radioimmunoassay, PGA is stable in tissue culture medium, at 37°C, for about 24 h. Titration curves for cell viability and virus infection were performed with increasing concentrations of prostaglandins (from 0.5 to 8 $\mu g\ ml^{-1}$) on CBL and tumour cell lines up to 3 weeks. A PGA concentration of 4 $\mu g\ ml^{-1}$ was found to be capable of suppressing the proliferation of infected CBL cultures 4–5 weeks p.i. without affecting the survival of non-infected CBL cultures. Therefore this concentration was used in all the experiments described. PGAs were added every 4 days, starting from the onset of the co-culture. In two separate experiments, PGAs were added twice only in the first 48 h of the CBL/MT-2 co-culture, to verify whether there could be some 'priming' effect on the immune response of CBL challenged with HTLV-I donor allogeneic tumour cells.

*Dot blot analysis for viral DNA and RNA*

Genomic DNA was extracted from CBL or MT-2 cells by the standard proteinase K method. RNA extraction was performed following the guanidine thiocyanate protocol (Chirgwin et al., 1979). For dot blots, 3 $\mu g$ DNA or RNA samples were spotted on nitrocellulose filters (Schleicher & Schüll, Dassel, FRG). DNA was denatured and neutralised before spotting according to Kafatos et al. (1979). RNA samples were dissolved in 1 volume $H_2O$ + 1 volume 20 × SSC solution + 1 volume formaldehyde, denatured by heating at 65°C for 10 min and spotted on filters previously equilibrated in 20 × SSC. All filters were air-dried and baked for 2 h at 80°C.

Hybridisation was performed by using a $^{32}P$-nick-translated probe corresponding to the SstI-SstI fragment of the HTLV-I genome, isolated from the pMT-2 plasmid (kindly given by R.C. Gallo). This 8.5 kb fragment accounts for almost the entire HTLV-I genome. Nitrocellulose filters were hybridised for about 20 h in 10 × Denhardt's solution, 4 × SET and 0.1% SDS as described by Graziani et al. (1987). Unspecific background was removed by washing with decreasing salt concentrations up to 1 × SET/0.1% SDS. Kodak X AR 5 films (Kodak Company, Rochester, USA) were used for autoradiography.

*DNA and RNA synthesis in CBL/MT-2 co-cultures*

$^3H$-thymidine or $^3H$-uridine incorporation were tested in blast cells during the first 4 weeks p.i. Cells (2 × $10^5$ CBL per well + 4 × $10^4$ irradiated MT-2 cells per well) were plated on day 0 in flat-bottomed 96-well microtitre tissue culture plates (Falcon), methyl-$^3H$-thymidine or $^3H$-uridine (Amersham International, Amersham, UK) was added at the concentration of 1 $\mu Ci$ per well and cells were harvested 18 h later by microtitre cell harvester (Titertek 530, Flow Lab, Irvine, UK). Samples were counted in a beta-scintillation counter (LKB, Bromma, Sweden) and mean c.p.m.s of quadruplicate groups were compared by *t* test analysis.

*Indirect immunofluorescence for the phenotype markers of CBL/MT-2 co-cultures*

Anti-Leu 2a (CD8), Leu 3a (CD4), Leu 4 (CD3), Leu M3 and IL-2 receptor (CD25) monoclonal antibodies were purchased by Beckton and Dickinson (Milan, Italy) and used in the indirect test for immunofluorescence under routine conditions. FITC-conjugated anti-mouse F(ab')IgG were purchased from Bio-Yeda (Rehovot, Israel) and anti-mouse IgM ($\mu$) from KPL (Gaithesburg, USA). Air dried samples were fixed for 20 min in ethanol/acetic acid (9:1) at −20°C, rehydrated by washing twice with cold PBS (phosphate buffered saline pH 7.2) and stored at 4°C after being covered with glycerol and cover slips. An average of 600 cells was scored for each duplicate sample and the percentages of positive cells were compared by $\chi^2$ analysis.

*Assay for the cell-mediated cytotoxicity of whole CBL or macrophages*

The natural killer (NK) activity of CBL was tested on day 0 against the NK-susceptible K562 target cells at graded effector/target cell ratios (E/T; 100:1, 50:1, 25:1 12.5:1) in a 4 h $^{51}Cr$-release assay under routine conditions (Graziani et al., 1987). CBL were pretreated overnight with 4 $\mu g\ ml^{-1}$ $PGA_1$ or di-M-$PGA_2$. The natural and antigen-specific cellular cytotoxicity of PGA-treated CBL, co-cultured with MT-2 cells, were respectively tested on day 7 p.i. against labelled K562 or MT-2 target cells in a 4 h $^{51}Cr$-release assay. In two separate experiments, cytotoxicity was evaluated on CBL subpopulations isolated by a double adherence step. Non-adherent cells (mostly lymphocytes, Ly) and adherent cells (mostly monocytes, Mo) were separately treated with PGA (4 $\mu g\ ml^{-1}$) and tested on days 0 and 7 for cytotoxicity against the NK-sensitive target K562 cells and the HTLV-I donor MT-2 cells in a 4 h $^{51}Cr$-release assay using 20:1, 10:1 and 5:1 E/T ratios. Alternatively, lymphocytes were infected by co-culture with irradiated MT-2 cells under standard conditions and added as inhibitory cells to autologous 7-day-old macrophages (M$\varphi$) (1:1 ratio) pretreated (or not) with 4 $\mu g\ ml^{-1}$ $PGA_1$ for 1 week, before testing for cell-mediated cytotoxicity against K562 or MT-2 targets.

Per cent cytotoxicity was calculated according to the formula:

$$\%\ \text{specific lysis} = \frac{\text{c.p.m. sample release} - \text{c.p.m. autologous release}}{\text{total c.p.m.}} \times 100$$

and dose–response curves were obtained by plotting the percentages of specific $^{51}Cr$-release of different E/T ratios (Thorn & Henney, 1976) and the number of killed cells (KC) per million effector cells was calculated. Significance (*P*) was calculated by regression test analysis.

### Results

*Effect of PGA treatment on* in vitro *infection of CBL with HTLV-I*

Non-stimulated human CBL can be kept in culture in the presence of IL-2, in the condition described in the Methods section, for 4–6 weeks. During the first week of culture, DNA synthesis of CBL, measured by $^3H$-thymidine incorporation, was very low when the cells were cultured in the absence of IL-2. When 20 U $ml^{-1}$ IL-2 were added to the medium, as routinely used for long-term lymphocyte cultures (Akagi *et al.*, 1985), isotope incorporation progressively increased and peaked in the first week of culture (data not shown). Graded amounts of PGAs (0.5–4 μg $ml^{-1}$) added to IL-2 supplemented CBL cultures every 4 days until the fourth week did not significantly decrease cell number and viability. However, the marked increase of $^3$H-thymidine incorporation induced by the presence of IL-2 and herein described was depressed by approximately 50% by $PGA_1$ (4 μg $ml^{-1}$) addition (data not shown). This discrepancy between cell number and thymidine incorporation would reflect a different balance between cell proliferation and death of selected CBL subpopulations under the influence of IL-2 alone or IL-2 + $PGA_1$. No early peak of $^3$H thymidine incorporation was detected when CBL were co-cultured with MT-2 cells, in spite of IL-2 supply (data not shown).

When cord-blood derived (HTLV-I + ) MT-2 cells were treated with PGAs, a dose-dependent inhibition of cell proliferation was obtained. A single PGA treatment (4 μg $ml^{-1}$) was effective in inhibiting MT-2 proliferation (seeded at a density of $10^5$ cells $ml^{-1}$ in 24-well plates, 2 ml per well) for 72 h, without significantly altering cell viability (at 72 h control $4.19 \pm 0.08 \times 10^5$ cells $ml^{-1}$; $PGA_1$ $1.65 \pm 0.15 \times 10^5$ cells $ml^{-1}$; viability 96%). $PGA_1$ concentrations lower than 1 μg $ml^{-1}$ had no effect, whereas concentrations higher than 10 μg $ml^{-1}$ completely prevented cell replication, but were toxic to the cells (viability decreased to 89, 59 and 50% with $PGA_1$ concentrations of 10, 15 and 30 μg $ml^{-1}$, respectively).

After co-culturing with MT-2 cells, CBL usually passed through a growth crisis within the first 2–3 weeks of co-culture. After this period, infected cells became predominant and, approximately 12 weeks p.i., apparently immortalised and occasionally independent of IL-2. Depending on different donors, the proliferation rate of infected CBL in some experiments increased already in the second week p.i., without going through a growth crisis.

Addition of 4 μg $ml^{-1}$ every 4 days of either $PGA_1$ or di-M-$PGA_2$ to CBL/MT-2 co-culture resulted in a remarkable dose-dependent inhibition of the late CBL proliferation and prevented the selection of immortalised HTLV-I + clones (Table I and Figure 1). In six separate CBL/MT-2 co-cultures tested, PGA-treated CBL survived for 4 weeks in the IL-2 enriched medium at levels comparable with normal non-infected controls and within the sixth week practically all cells died as expected for normal CBL (Table I). The kinetics

**Figure 1** Effect of PGA treatment on the growth (viable cells $ml^{-1}$) of CBL/MT-2 cocultures (○——○). $PGA_1$ (4 μg $ml^{-1}$, □——□) or di-M-$PGA_2$ (4 μg $ml^{-1}$, △——△) were added to the CBL/MT-2 cocultures following a multiple treatment scheme (every 4 days, **a**) or a short-term treatment at the onset of the coculture (day 0 and 2, **b**). ●——●, non-infected CBL cultures (s.e. within 10% of the mean for quadruplicate samples).

of $^3$H-thymidine incorporation paralleled the patterns of cell growth, showing no inhibition exerted by PGAs on the minimal proliferation rate of CBL/MT-2 co-cultures within the first week p.i. and a clear-cut inhibition of late CBL proliferation, starting 2 weeks p.i. and resulting in a marked suppression of $^3$H incorporation 4 weeks p.i. (Figure 2).

The effect of PGAs was strictly dependent on multiple treatments. In fact, when PGAs were added only at the onset of the co-culture (time 0) or in the first 2 days (time 0 and

**Table I** Time-course of cell growth (viable cells $ml^{-1}$) of CBL co-cultured with irradiated MT-2 (HTLV-I donor) cells after multiple treatments with $PGA_1$ or di-M-$PGA_2$ (4 μg $ml^{-1}$ every 4 days)

| | *Viable cells × $10^5$ $ml^{-1}$* | | | | |
|---|---|---|---|---|---|
| | *1 week* | *2 weeks* | *3 weeks* | *4 weeks* | *6 weeks* |
| Experiment 1 | | | | | |
| CBL/MT-2 | 4.7 | 10.1 | 14.0 | 14.0 | 10.0 |
| CBL/MT-2 + $PGA_1$ | 3.8 | 3.0 | 1.0 | 0.5 | 0.0 |
| CBL/MT-2 + di-M-$PGA_2$ | 2.1 | 0.8 | 6.0 | 0.6 | 0.0 |
| Experiment 2 | | | | | |
| CBL/MT-2 | 3.8 | 6.8 | 6.8 | n.t. | 9.3 |
| CBL/MT-2 + $PGA_1$ | 2.8 | 7.3 | 3.9 | n.t. | 0.0 |
| CBL/MT-2 + di-M-$PGA_2$ | 3.1 | 5.0 | 6.0 | n.t. | 0.0 |
| Experiment 3 | | | | | |
| CBL/MT-2 | 2.8 | 1.2 | 1.3 | 3.6 | 7.2 |
| CBL/MT-2 + $PGA_1$ | 3.2 | 1.0 | 1.9 | 0.5 | 0.0 |
| Experiment 4 | | | | | |
| CBL/MT-2 | 12.4 | n.t. | n.t. | 1.5 | n.t. |
| CBL/MT-2 + $PGA_1$ | 5.3 | n.t. | n.t. | 0.7 | n.t. |
| Experiment 5 | | | | | |
| CBL/MT-2 | 3.4 | 4.2 | 6.4 | 5.5 | 9.8 |
| CBL/MT-2 + $PGA_1$ | 6.6 | 1.6 | 1.2 | 0.7 | 0.1 |
| Experiment 6 | | | | | |
| CBL/MT-2 | 1.2 | 0.8 | 0.2 | 2.2 | 7.9 |
| CBL/MT-2 + di-M-$PGA_2$ | 1.2 | 0.4 | 0.0 | 0.0 | 0.0 |

n.t., not tested.

**Figure 2** Effect of multiple treatments with $PGA_1$ (4 µg $ml^{-1}$) on the proliferation rate ($^3$H-thymidine incorporation) of CBL/MT-2 co-cultures. ○——○, untreated CBL/MT-2 co-culture; □——□, $PGA_1$ treated co-culture (s.e. within 10% of the mean for quadruplicate samples). The viable cell numbers at 4 weeks p.i. were $6 \times 10^5$ $ml^{-1}$ in the CBL/MT-2 culture and $0.7 \times 10^5$ $ml^{-1}$ in the PGA-treated co-culture.

48 h) of co-culture, late CBL proliferation was not impaired. Moreover, in one experiment (shown in Figure 1) CBL proliferation was even enhanced by a single PGA treatment and the growth of HTLV-I infected cells appeared actually to be favoured since PGA-treated CBL/MT-2 contained 35% p19 + cells as compared to 10% present in untreated CBL/MT-2 co-cultures at 8 weeks p.i.

Repeated PGA treatments either did not affect or increased the percentage of p19 + CBL during the first 2 weeks p.i. (Table II). At later stages of infection (i.e. 4 weeks p.i.) the proportion of p19 + cells in PGA-treated co-cultures was comparable to or lower than in untreated controls. In any case, even in the co-cultures in which p19 expression was increased at 2 weeks p.i., the anti-proliferative effect of repeated PGAs treatment on HTLV-I infected cells was confirmed, since 'immortalised' HTLV-I + clones were detected in controls but not in PGA-treated CBL/MT-2 co-cultures.

### *HTLV-I integration and expression in CBL/MT-2 co-cultures after PGA-treatment*

The results obtained by dot blot analysis on genomic DNA extracted from co-cultured CBL showed comparable amounts of integrated provirus in PGA-treated and untreated CBL, 2 weeks p.i. (Figure 3). Four weeks p.i., no integrated viral DNA was detectable in most PGA-treated CBL/MT-2 cell preparations (data not shown). When virus expression was tested, it was found that the amount of viral RNA was greatly augmented in infected CBL after PGA-treatment, 2 weeks p.i., approaching the amount detected in the 100% p19 + MT-2 cells. No difference in the percentage of p19 + cells was instead found between PGA-treated and untreated CBL in the same experiment (Figure 3). However, to this enhancement of HTLV-I mRNA in treated CBL there was no corresponding increase of total RNA synthesis, since no significant difference in $^3$H-uridine incorporation was found when control CBL, CBLMT-2 co-cultures or CBL/MT-2 PGA-treated co-cultures were compared, up to the third week of co-culture (data not shown).

### *Expression of phenotype markers in PGA-treated CBL/MT2 co-cultures*

Data are summarised in Table II. A clear-cut picture can be observed when non-infected CBL cultures are compared to CBL/MT-2 co-cultures. Four days p.i., a general suppression of phenotypic markers was noted in CBL/MT-2 co-cultures, with the exception of a 3–4-fold increase of M3 phenotype, which identifies monocyte/macrophage subsets. PGAs increased the relative percentage of CD3 + and M3 + cells in non-infected CBL cultures. No variation of CD3 marker was observed in infected CBL following PGA treatment, whereas M3 phenotype was significantly reduced. It follows that PGAs would differently regulate monocyte proliferation in CBL cultures when these are non-infected, as compared to cultures exposed to HTLV-I infection.

No significant variation in the expression of IL-2 receptor was found at early stages of HTLV-I infection and PGAs did not further affect it (Table III). As expected, however, the immortalised clones that arised from untreated CBL/MT-2 co-cultures, tested 12 weeks p.i., expressed high IL-2 receptor and CD4 phenotype (data not shown).

**Figure 3** Dot blot analysis of genomic DNA or mRNA samples (3 µg per spot) showing integration of HTLV-I provirus and its expression in CBL/MT-2 co-cultures 15 days p.i., in the presence or the absence of $PGA_1$ (4 µg $ml^{-1}$). Per cent p19 + cells are reported in the third lane. CBL are negative control samples and MT-2 cells are 100% positive for p19 protein.

**Table II** Time-course of p19 positivity in CBL co-cultured with irradiated MT-2 (HTLV-I donor) cells after multiple treatments with $PGA_1$ (4 µg $ml^{-1}$ every 4 days)

| | *1 week* | | *2 weeks* | | *4 weeks* | |
|---|---|---|---|---|---|---|
| *Cells* | *%p19 +* | *P* | *%p19 +* | *P* | *%p19 +* | *P* |
| Experiment 1 | | | | | | |
| CBL/MT-2 | 6.35 | – | 13.07 | – | 7.01 | – |
| CBL/MT-2 + $PGA_1$ | 7.92 | n.s. | 27.51 | <0.01 | 8.48 | n.s. |
| Experiment 2 | | | | | | |
| CBL/MT-2 | 4.32 | – | 6.18 | – | 29.8 | – |
| CBL/MT-2 + $PGA_1$ | 6.47 | n.s. | 5.64 | n.s. | 10.7 | <0.01 |
| Experiment 3 | | | | | | |
| CBL/MT-2 | 14.32 | – | 8.1 | – | 2.95 | – |
| CBL/MT-2 + $PGA_1$ | 13.42 | n.s. | 8.3 | n.s. | 4.77 | n.s. |

*P*, probability, calculated by $\chi^2$ analysis.

**Table III** Variation in the expression of phenotypic markers in CBL/MT-2 co-cultures as compared to non-infected CBL and its modulation by PGAs

| Sample | Drug | Leu 2a(CD8) % | Leu 2a(CD8) P | Leu 3a(CD4) % | Leu 3a(CD4) P | Leu 4(CD3) % | Leu 4(CD3) P | IL-2 receptor (CD25) % | IL-2 receptor (CD25) P | Leu M3(CD14) % | Leu M3(CD14) P |
|---|---|---|---|---|---|---|---|---|---|---|---|
| CBL | – | 30.86 | – | 27.25 | – | 48.90 | – | 9.45 | – | 6.12 | – |
| CBL | $PGA_1$ | 29.34 | n.s. | 28.51 | n.s. | 51.79 | n.s. | 8.53 | n.s. | 12.13 | <0.01 |
| CBL | di-M-$PGA_2$ | 29.52 | n.s. | 33.74 | <0.05 | 62.54 | <0.01 | 8.03 | n.s. | 10.55 | <0.01 |
| CBL/MT-2 | – | 5.85 | – | 3.45 | – | 1.54 | – | 11.58 | – | 22.77 | – |
| CBL/MT-2 | $PGA_1$ | 7.14 | n.s. | 3.18 | n.s. | 1.07 | n.s. | 14.72 | n.s. | 16.41 | <0.01 |
| CBL/MT-2 | di-M-$PGA_2$ | 8.31 | n.s. | 3.48 | n.s. | 1.25 | n.s. | 13.12 | n.s. | 13.24 | <0.01 |

PGAs ($4 \mu g\ ml^{-1}$) were added on day 0 and 3, cells were then harvested on day 4 and tested with the monoclonal antibody panel. Expression of CD8, CD4 and CD3 markers was significantly decreased after co-culture of CBL with MT-2 cells ($P<0.01$), whereas the percentage of cells expressing IL-2 receptor slightly increased and M3 subpopulation was significantly expanded ($P<0.01$). $P$ was calculated by $\chi^2$ analysis.

### *Cell-mediated immunity in PGA-treated CBL/MT-2 co-cultures*

PGs are well known for their modulating activity on the immune response. Therefore studies of cell-mediated immunity were performed in terms of: (a) cell-mediated natural cytotoxicity against NK-susceptible K562 or relatively NK-insensitive MT-2 target cells; (b) cytotoxic T lymphocyte (CTL) response against MT-2 'sensitiser' cells; (c) macrophage-mediated cytotoxicity.

Freshly isolated CBL were incubated overnight at 37°C with medium alone, or with medium containing $4 \mu g\ ml^{-1}$ of $PGA_1$ or di-M-$PGA_2$, washed and tested for NK activity against K562 or MT-2 target cells. The results illustrated in Figure 4 show that the cytotoxicity of effector cells against K562 targets was significantly reduced by PGAs. The cytolytic activity of CBL was marginal against MT-2 cells and PGAs did not show any detectable effect.

CBL co-cultured with irradiated MT-2 cells in IL-2 enriched medium were tested for cytotoxicity against K562 and MT-2 targets on day 7 p.i. The results show that: (a) the cytolytic activity of CBL/MT-2 effectors against K562 or MT-2 cells was respectively 40% or 87% lower than non-infected controls (Figure 5, legend); (b) PGAs further inhibited the killing capacity of infected CBL against K562 targets, but slightly enhanced their cytotoxic activity against MT-2 target cells (Figure 5).

Cord blood monocytes showed marginal cytotoxic activity against both K562 and MT-2 target cells when tested on day 0 in a 4 h $^{51}Cr$-release assay (Figure 6). After 1 week of culture, monocytes differentiated into macrophages (Mφ) and acquired most functions of mature resident Mφ (D'Onofrio & Lohmann-Matthes, 1984; D'Onofrio & Paradisi, 1983).

**Figure 4** Natural killing (NK) activity of freshly isolated CBL against the NK-susceptible target K562 cells and its negative-modulation by PGAs ($P<0.01$, calculated by regression analysis). On the right side, the modest baseline killing capacity of fresh CBL against MT-2 cells (expressing class I and II antigens) is shown not to be affected by PGA treatment.

**Figure 5** Effect of PGA treatment on the cell-mediated cytotoxicity of CBL/MT-2 co-cultured effector cells, tested on day 7 p.i. against the NK-susceptible target K562 cells and against MT-2 cells. PGAs significantly reduced the 'anomalous killing' of CBL against K562 target ($P<0.01$, calculated by regression analysis), whereas they did not affect the killing of infected CBL against MT-2 targets. Infection with HTLV-I (CBL/MT-2 co-cultures, grown in medium supplemented with $20\ U\ ml^{-1}$ IL-2, tested 7 days p.i.), resulted in a clear-cut depression of CBL killing ability (number of killed K562 target cells by CBL = 4,022, versus 2,396 killed cells by CBL MT-2 co-cultures, $P<0.01$; number of killed MT-2 target cells by CBL = 1,840, versus 228 killed cells by CBL/MT-2 effectors, $P<0.01$).

Their cytotoxic capacity against both K562 and MT-2 targets increased markedly and was not affected by PGA treatment (Figure 6). Autologous non-adherent mononuclear cells (mostly T lymphocytes), cultured in IL-2 enriched medium for 1 week, showed higher killing activity against both targets as compared to Mφ (see Figure 6, legend). In this case PGA treatment of isolated lymphocytes profoundly inhibited their killing activity against K562 targets (data not shown). When 7-day cultured non-adherent cells (i.e. Ly, see Figure 6) where admixed with separately cultured Mφ at 1:1 ratio and used as effector cells in the cytotoxicity assay against both K562 or MT-2 targets, the results (Figure 6) showed that: (a) additive cytotoxic effects of Mφ + Ly were detected against K562 cells, whereas antagonistic effects were found when MT-2 targets were used; in this case the cytolytic effects were less than the sum of Ly and Mφ cytotoxicity, and did not exceed that of Ly alone; (b) the killer activity of Mφ + Ly against K562 or MT-2 cells was significantly inhibited by $PGA_1$ treatment of Mφ on day 0, 3 and 6; (c) when Ly exposed to HTLV-I infection (i.e. Ly/MT-2) were admixed with Mφ, antagonistic effects on the cytolytic activity against both K562 and MT-2 cells were detected; this was demonstrated by the finding that in no case did the cytotoxicity of Mφ + Ly/MT-2 exceed that of Mφ alone, in spite of a substantial lytic activity of Ly/MT-2 against K562 or MT-2 cells as well (see Figure 6, legend); (d) pretreatment of Mφ with

**Figure 6** Effect of PGA treatment on monocyte (day 0) and macrophage (Mφ, day 7) cytotoxicity against NK-susceptible K562 targets and against MT-2 (HTLV-I +) cells. Mφ were treated with 4 μg ml$^{-1}$ $PGA_1$ (hatched bars) on days 0, 3 and 6 tested for cytotoxicity on day 7 in a short-time $^{51}Cr$-release assay (4 h, as for lymphocyte test). Autologous lymphocytes (Ly) were added to Mφ cultures at 1:1 ratio just at the time of the test, and the resulting killing capacity was compared by testing non-infected or HTLV-I infected Ly (i.e. 7-day-old autologous Ly/MT-2 co-cultures grown in medium supplemented with 20 U ml$^{-1}$ IL-2). The numbers of killed target cells in different conditions are also reported. *n* killed K562 cells by: whole CBL = 4,022; Ly = 20,128; Mφ = 6,824; Ly + Mφ = 29,760 (in Figure). *n* killed MT-2 cells by: whole CBL = 1,840; Ly = 16,504; Mφ = 7,368; Ly + Mφ = 16,336 (in Figure). *n* killed K562 targets by: whole CBL/MT-2 co-cultures = 2,396; Ly/MT-2 = 5,704; (Ly/MT-2) + Mφ = 10,000 (in Figure). *n* killed MT-2 targets by: whole CBL/MT-2 co-cultures = 228; Ly/MT-2 = 6,552; (Ly/MT-2) + Mφ = 7,200 (in Figure).

PGAs did not modify the cytotoxic activity of these cells admixed with Ly exposed to HTLV-I infection, tested against K562 or MT-2 targets.

### Discussion

Infection with HTLV-I, as for other leukaemia viruses, is usually followed by initial target cell proliferation, growth crisis and virus-induced polyclonal hyperplasia of infected cells that might undergo monoclonal selection and be immortalised *in vitro* (Hahn *et al.*, 1984) or associate with ATL leukaemia *in vivo* after a variably long latency period (Hahn *et al.*, 1984; Yoshida, 1987). This early virus-induced proliferation of sensitive target cells can be considered a predisposing condition to stable transformation (Duesberg, 1987), according with a multistep model of tumorigenesis and under the control of specific host genes (Knudson, 1985). Hence, a predictable tumour risk would depend on high virus expression and virus-induced hyperplasia. Prevention of this risk is reasonably focused on the possibility of affecting these two steps of retrovirus pathogenicity.

Interferon-β was recently shown to inhibit HTLV-I transmission and integration in specific target cells, resulting in a sharp decrease in the number of p19 + lymphocytes during early weeks p.i. (D'Onofrio *et al.*, 1988). However, early interferon treatment of lymphocytes delayed the HTLV-I induced hyperplasia, but did not prevent it. In contrast, when type A prostaglandins were tested, no clear-cut inhibition of HTLV-I infection was found 2–3 weeks p.i., but later the virus-induced proliferation of lymphocytes appeared to be greatly impaired by multiple PGA treatments, so that no selection of HTLV-I infected clones apparently occurred. Both $PGA_1$ and di-M-$PGA_2$ were highly effective in preventing the HTLV-I-induced hyperplasia when used at the concentration of 4 μg ml$^{-1}$. PGAs also inhibited the proliferation of the HTLV-I + MT-2 cells, an established T-cell line derived from infected CBL (Miyoshi *et al.*, 1981). On the contrary, after a single treatment the prevalent effect of PGAs on CBL/MT-2 co-cultures was an enhancement of infection and the late proliferative phase was not impaired.

Multiple mechanisms of action of prostaglandins may contribute to the final results described here. A possible effect on the replicative cycle of HTLV-I was first hypothesised, because of the potent anti-viral activity of PGAs demonstrated in several DNA and RNA virus models and associated with inhibition of specific virus protein synthesis (usually 'late' proteins or glycoproteins) and block of virus maturation (Santoro, 1987; Santoro *et al.*, 1987). However, there is no evidence that PGAs could inhibit replication of oncogenic viruses. On the contrary, several prostaglandins ($PGA_1$, $PGB_2$ $PGE_2$ and $PGF_{2\alpha}$) have been reported to enhance replication of murine mammary adenocarcinoma virus (Karmali *et al.*, 1982; Svec *et al.*, 1982) and of HIV (by $PGE_2$; Ueno *et al.*, 1987). In our system, PGAs were found not to decrease and in some cases even to enhance the degree of CBL infection during the first 2 weeks p.i., as determined by the percentage of p19 + cells and by the amount of cytoplasmic viral mRNA. Studies are in progress to define whether PGAs affected transcription from integrated provirus or stabilisation of viral RNA. Moreover, a possible action of PGAs on the synthesis and maturation of HTLV-I proteins has also to be verified.

The ability of multiple PGA-treatments to prevent HTLV-I induced hyperplasia and the selection of leukaemic clones could be due to a direct anti-proliferative effect of PGAs. In fact, they have been shown to inhibit cell proliferation and/or promote differentiation in several leukaemic mouse and human cell lines; this activity was found to be dependent on the stage of cell differentiation and on the phase of the cell cycle, and usually was not related to cAMP metabolism (Santoro, 1987; Santoro *et al.*, 1986; Santoro & Jaffe, 1989). In particular, the antiproliferative effect of PGAs was dose-dependent, reversible depending on the length of treatment and associated with inhibition of protein synthesis and glycosylation (Santoro, 1987; Santoro *et al.*, 1986).

HTLV-I infection is followed *in vitro* and *in vivo* by remarkable alteration of the immune response (De Vecchis *et al.*, 1985; D'Onofrio *et al.*, 1988; Sociu-Foca *et al.*, 1984; Volkman *et al.*, 1985; Yarchoan *et al.*, 1986) and it has been suggested that the indiscriminant helper function of infected T lymphocytes plays a major role in the auto-maintenance of the leukaemic state (Volkman *et al.*, 1985). These observations suggested the possibility that the effect of PGAs described could be mediated by interaction with the host's immune system challenged by exposure to the virus. The *in vitro* and possibly *in vivo* pattern of the first step of HTLV-I infection implies a cell-to-cell virus transmission accompanied by donor–recipient allogeneic interaction, which elicits a T-cell mediated immune response that in turn could restrain HTLV-I infection. Following this initial step, elimination of autologous virus infected cells is strictly dependent on the efficiency of killing by effector monocytic and lymphocytic cells.

The effects of PGAs on the whole CBL population or on Ly and Mφ effector cells were analysed separately to ascertain whether PGAs could be involved in ultimate virus suppression mediated by the killing of virus-infected cells. Actually PGA treatment did not increase the spontaneous cytotoxicity of cultured Mφ or Mφ + Ly, or the CTL-mediated cytolysis of supposedly allosensitised effector cells (i.e.

Mφ + (Ly/MT-2), against MT-2 targets. This finding does not support the hypothesis that immune mechanisms would play a key role in the PGA-induced suppression of the outgrowth of HTLV-I infected clones, potentially capable of inducing CTL responses (Mitsuya *et al.*, 1983). The transient but remarkable depression of the majority of lymphocyte markers except for M3 antigens in CBL exposed to virus infection is difficult to interpret. However, this occurrence might contribute to an explanation of the failure of potential immunosurveillance mechanisms to control the emergence of the infected clone. The influence of PGA treatment on these parameters was modest, except for M3. In this case PGAs increased or decreased the percentage of M3 positive cells in cultured control CBL or CBL/MT-2 respectively. Studies are in progress to elucidate the role of Mφ in the early phase of infection.

In conclusion, the present data suggest that PGAs could play a significant role in controlling HTLV-I infection by non-immunological mechanisms. Further studies are in progress to explore whether PGA treatment combined with immunomodulating agents (i.e. agents capable of antagonising the immune depression that follows the infection with HTLV-I) would improve the efficiency of *in vitro* control of infection, in the attempt to elaborate experimental protocols for prevention of HTLV-I related disease in seropositive subjects.

We are grateful to Dr R.C. Gallo for kindly providing us with pMT-2 plasmid and to Dr M. Robert-Guroff for anti-p19 monoclonal antibody. We wish to thank Mrs G. Trapella and Miss M.C. Mastrilli for helpful editing assistance. This work was supported by CNR, 'PF Oncologia' (contract no. 104348) and CNR-National Science Foundation USA-Italy Cooperative Program, grant no. 8803273.

### References

AKAGI, T., OHTSUKI, Y., TAKAHASHI, K., TAKEDA, I., OKA, T. & MIYOSHI, I. (1985). Immortalization of human lymphocytes by co-cultivation with lethally irradiated autologous T-cell lines harbouring HTLV-I. *J. Cancer Res. Clin. Oncol.*, **110**, 82.

BAILEY, J.M. & FLETCHER-CIENTAT, M. (1987). Prostaglandins and leukotrienes in T-helper and T-suppressor cell system. In *Prostaglandins in Cancer Research*, Garaci, E., Paoletti, R. & Santoro M.G. (eds) p. 202. Springer-Verlag: Berlin.

BONTA, I.L. & BEN-EFRAIM, S. (1987). Leukotrienes and prostaglandins mutually govern the antitumor potential of macrophages. In *Prostaglandins in Cancer Research*, Garaci, E., Paoletti, R. & Santoro M.G. (eds) p. 193. Springer-Verlag: Berlin.

CHIRGWIN, J.M., PRZYBYLA, A.E., MACDONALD, R.J. & RUTTER, W.J. (1979). Isolation of biologically active ribonucleic acid from sources enriched in ribonuclease. *Biochemistry*, **18**, 5294.

DE VECCHIS, L., GRAZIANI, G., MACCHI, B. & 5 others (1985). Decline of natural cytotoxicity of human lymphocytes following infection with human T-cell leukemia/lymphoma virus (HTLV). *Leuk. Res.*, **9**, 349.

D'ONOFRIO, C. & LOHMANN-MATTHES, M.L. (1984). Chemiluminescence of macrophages depends upon their differentiation stage: dissociation between phagocytosis and oxygen radical release. *Immunobiology*, **167**, 414.

D'ONOFRIO, C. & PARADISI, F. (1983). *In vitro* differentiation of human monocytes into mature macrophages during long-term culture. *Immunobiology*, **164**, 13.

D'ONOFRIO, C., PERNO, C.F., MAZZETTI, P., GRAZIANI, G., CALIO, R. & BONMASSAR, E. (1988). Depression of early phase of HTLV-I infection *in vitro* mediated by human beta-interferon. *Br. J. Cancer*, **57**, 481.

DUESBERG, P.H. (1987). Retroviruses as carcinogens and pathogens: expectation and reality. *Cancer Res.*, **47**, 1199.

FRANCHINI, F., WONG-STAAL, F. & GALLO, R.C. (1984). Human T-cell leukemia virus (HTLV-I) transcripts in fresh and cultured cells of patients with adult T-cell leukemia. *Proc. Natl Acad. Sci. USA*, **81**, 6207.

GALLO, R.C. (1985). The human T-cell leukemia/lymphotropic retrovirus (HTLV) family: past, present and future. *Cancer Res.*, **45**, 4524s.

GRAZIANI, G., PASQUALETTI, M., LOPEZ, M. & 5 others (1987). Increased susceptibility of peripheral mononuclear cells of leukemic patients to HTLV-I infection *in vitro*. *Blood*, **69**, 1175.

HAHN, B., GALLO, R.C., FRANCHINI, F. & 5 others (1984). Clonal selection of human T-cell leukemia virus-infected cells *in vivo* and *in vitro*. *Mol. Biol. Med.*, **2**, 29.

KAFATOS, F.C., WELDON-JONES, C. & EFSTRATIADIS, A. (1979). Determination of nucleic acid sequence homologies and relative concentrations by a dot hybridization procedure. *Nucleic Acid Res.*, **7**, 1541.

KARMALI, R.A., SARKAR, N.H., WHITTINGTON, E. & GOOD, R.A. (1982). Prostaglandin regulation of murine mammary tumor virus production: a basis for some of the glucocorticoid and prolactin action on mammary tumor cell cultures. *Prostaglandin Leukotriene Med.*, **9**, 641.

KNUDSON, A.J. (1985). Hereditary cancer, oncogenes and anti-oncogenes. *Cancer Res.*, **45**, 1437.

LOZZIO, C.B. & LOZZIO, B.B. (1975). Human chronic myelogenous leukemia cell line with positive Philadelphia chromosome. *Blood*, **45**, 321.

MANZARI, V., GRADILONE, A., BARILLARI, G. & 8 others (1985). HTLV-I is endemic in Southern Italy: detection of the first cluster of infection in a white population. *Int. J. Cancer*, **36**, 557.

MITSUYA, H., MATIS, L.A., MEGSON, M. & 5 others (1983). Generation of an HLA-restricted cytotoxic T-cell line reactive against cultured cells from a patient infected with human T-cell leukemia/lymphoma virus (HTLV). *J. Exp. Med.*, **158**, 994.

MIYOSHI, I., KUBONISHI, I., YOSHIMOTO, S. & 5 others (1981). Type C particles in a cord T-cell line derived by cocultivating normal human cord leukocytes and human leukemic T-cells. *Nature*, **296**, 770.

OLSSON, I.L., BREITMAN, T.R. & GALLO, R.C. (1982). Priming of human myeloid leukemia cell lines HL-60 and U937 with retinoic acid for differentiation effects of cyclic adenosine 3′:5′-monophosphate-inducing agents and a T-lymphocyte-derived differentiation factor. *Cancer Res.*, **42**, 3928.

ROBERT-GUROFF, M., RUSCETTI, F.W., POSNER, L.E., POIESZ, B.J. & GALLO, R.C. (1981). Detection of the human T-cell lymphoma virus p19 in cells of some patients with cutaneous T-cell lymphoma and leukemia using a monoclonal antibody. *J. Exp. Med.*, **154**, 1957.

SANTORO, M.G. (1987). Involvement of protein synthesis in the antiproliferative and antiviral action of prostaglandins, In *Prostaglandins in Cancer Research*, Garaci, E., Paoletti, R. & Santoro, M.G. (eds) p. 97. Springer-Verlag: Berlin.

SANTORO, M.G., BENEDETTO, A., CARRUBA, G., GARACI, E. & JAFFE, B.M. (1980). Prostaglandin A compounds as antiviral agents. *Science*, **209**, 1032.

SANTORO, M.G., CARRUBA, G., GARACI, E., JAFFE, B.M. & BENEDETTO, A. (1981). Prostaglandins of the A series inhibit Sendai virus replication in cultured cells. *J. Gen. Virol.*, **53**, 75.

SANTORO, M.G., CRISARI, A., BENEDETTO, A. & AMICI, C. (1986). Modulation of the growth of a human erythroleukemic cell line (K562) by prostaglandins: antiproliferative action of PGAs. *Cancer Res.*, **46**, 6073.

SANTORO, M.G., FAVALLI, C., MASTINO, A., JAFFE, B.M., ESTEBAN, M. & GARACI, E. (1988). Antiviral activity of a synthetic analog of prostaglandin A in mice infected with influenza A virus. *Arch. Virol.*, **99**, 89.

SANTORO, M.G., FUKUSHIMA, M., BENEDETTO, A. & AMICI, C. (1987). $PGJ_2$, a new antiviral prostaglandin: inhibition of Sendai virus replication and alteration of virus protein synthesis. *J. Gen. Virol.*, **68**, 1153.

SANTORO, M.G. & JAFFE, B.M. (1989). Prostaglandins and differentiation of Friend erythroleukemia cells. In *Prostaglandins and Tumor Cell Proliferation and Differentiation*, Hammarstrom, S. (ed). M. Nijhoff: The Hague.

SOCIU-FOCA, N., RUBINSTEIN, P., POPOVIC, M., GALLO, R.C. & KING, D.W. (1984). Reactivity of HTLV-transformed human T-cell lines to MHC class II antigens. *Nature*, **312**, 275.

SVEC, J., SVEC, P., HALCAK, L. & THURZO, V. (1982). Role of natural prostaglandins in the control of murine mammary tumor expression. *J. Cancer Res. Clin. Oncol.*, **103**, 55.

THORN, R.M. & HENNEY, C.S. (1976). Kinetic analysis of target cell destruction by effector T-cells: I. Delineation of parameters related to the frequency and lytic efficiency of killer cells. *J. Immunol.*, **117**, 2213.

UENO, R., KUNO, S. & HAYAISHI, O. (1987). Prostaglandin $E_2$, a seminal constituent, facilitates the replication of acquired immuno-deficiency syndrome (AIDS) virus *in vitro*. In *Prostaglandins in Cancer Research*, Garaci, E., Paoletti, R. & Santoro, M.G. (eds) p. 277. Springer-Verlag: Berlin.

VOLKMAN, D.J., POPOVIC, M., GALLO, R.C. & FAUCI, A.S. (1985). Human T-cell leukemia/lymphoma virus-infected antigen-specific T-cell clones: indiscriminant helper function and lymphokine production. *J. Immunol.*, **134**, 4237.

YOSHIDA, M. (1987). Expression of the HTLV-I genome and its association with a unique T-cell malignancy. *Biochim. Biophys. Acta*, **907**, 145.

YARCHOAN, R., GUO, H.G., REITZ, M.S., MALUISH, A., MITSUYA, H. & BRODER, S. (1986). Alterations in cytotoxic and helper T-cell function after infection of T-cell clones with human T-cell leukemia virus Type I. *J. Clin. Invest.*, **77**, 1466.

WONG-STAAL, F. & GALLO, R.C. (1985). Human T-lymphotropic retroviruses. *Nature*, **317**, 395.